# Application of Li_6.4_La_3_Zr_1.45_Ta_0.5_Mo_0.05_O_12_/PEO Composite Solid Electrolyte in High-Performance Lithium Batteries

**DOI:** 10.3390/ma17133094

**Published:** 2024-06-24

**Authors:** Chengjun Lin, Yaoyi Huang, Dingrong Deng, Haiji Xiong, Bin Lu, Jianchun Weng, Xiaohong Fan, Guifang Li, Ye Zeng, Yi Li, Qihui Wu

**Affiliations:** 1 College of Marine Equipment and Mechanical Engineering and Xiamen Key Lab of Marine Corrosion and Smart Protective Materials, Jimei University, Xiamen 361021, China; lcj15779775914@163.com (C.L.); xionghj1999@163.com (H.X.); lb5868933@163.com (B.L.); gingerwjc@163.com (J.W.); echo_fan@jmu.edu.cn (X.F.); guifangli1991@163.com (G.L.); zengye@jmu.edu.cn (Y.Z.); qihui.wu@jmu.edu.cn (Q.W.); 2 College of Resources and Environmental Sciences, Quanzhou Normal University, Quanzhou 362000, China; 3 Jiangsu Key Lab of Advanced Functional Polymer Design and Application, Department of Polymer Science and Engineering, College of Chemistry, Chemical Engineering and Materials Science, Soochow University, Suzhou 215125, China; liyi@suda.edu.cn

**Keywords:** solid lithium batteries, composite electrolyte, polyethylene oxide, LLZTMO

## Abstract

Replacing the flammable liquid electrolytes with solid ones has been considered to be the most effective way to improve the safety of the lithium batteries. However, the solid electrolytes often suffer from low ionic conductivity and poor rate capability due to their relatively stable molecular/atomic architectures. In this study, we report a composite solid electrolyte, in which polyethylene oxide (PEO) is the matrix and Li_6.4_La_3_Zr_1.45_Ta_0.5_Mo_0.05_O_12_ (LLZTMO) and Li_6.4_La_3_Zr_1.4_Ta_0.6_O_12_ (LLZTO) are the fillers. Ta/Mo co-doping can further promote the ion transport capacity in the electrolyte. The synthesized composite electrolytes exhibit high thermal stability (up to 413 °C) and good ionic conductivity (LLZTMO–PEO 2.00 × 10^−4^ S·cm^−1^, LLZTO–PEO 1.53 × 10^−4^ S·cm^−1^) at 35 °C. Compared with a pure PEO electrolyte, whose ionic conductivity is in the range of 10^−7^~10^−6^ S·cm^−1^, the ionic conductivity of composite solid electrolytes is greatly improved. The full cell assembled with LiFePO_4_ as the positive electrode exhibits excellent rate performance and good cycling stability, indicating that prepared solid electrolytes have great potential applications in lithium batteries.

## 1. Introduction

Rechargeable lithium batteries have become the most attractive electrochemical energy storage devices due to their high energy density [1], high power density [2], and long cycling life [3]. As for their applications in electrical (or hybrid) vehicles, the further promotion of energy density and safety of lithium batteries have been considered as the most crucial targets that needs to be achieved [4,5]. So far, considerable efforts have been made to substitute the commonly used carbon anodes with lithium metal [6], as lithium has a theoretical capacity (3860 mAh g^−1^) approximately one order of magnitude higher than graphite anodes (372 mAh g^−1^) [7,8]. In addition, the application of lithium metal can further increase the battery potential [9], thus enhancing the energy density. However, the practical applications of lithium metal as an anode in commercial batteries is limited by two significant safety issues: the formation and proliferation of lithium dendrites during charging [10], and the flammability and leakage of traditional organic liquid electrolytes [11,12]. In particular, lithium dendrites may accelerate the harmful reactions with the electrolytes [13], and even cause catastrophic battery failure due to internal short circuits when they penetrate the separator and finally connect to the cathode [14]. To overcome this, several strategies have been proposed to suppress the growth of lithium dendrites. One of them is to grow a predetermined solid electrolyte interface (SEI) on a lithium anode in situ by introducing additives [15,16]; another method is to design new anode material structures, for example, using lithophile graphene [17], nickel foam [18], and polyimide [19], to inhibit the growth of dendrites and at the same time reduce the huge volume change during charging/discharging. It is well known that solid electrolytes have the potential to mechanically inhibit the growth of lithium dendrites [20,21]. However, the most commonly studied solid electrolyte materials, such as polymers and ceramics, still have some drawbacks when practically applied in batteries at room temperature (25 °C) [22], including insufficient ion conductivity [8] and high electrode/electrolyte interfacial impedance [23,24]. Some sulfide-based [25] and garnet-type [26] solid electrolytes have ionic conductivity comparable to the liquid electrolytes, but the growth of lithium dendrites through the grain boundaries still remains a major issue [27]. Therefore, it is difficult to develop solid electrolytes that can completely block the growth of lithium branches [28]. In this case, it is essential to find a proper way to prepare solid electrolytes [29] in order to replace the flammable organic electrolytes [30,31], to greatly improve the safety of batteries [32]. Fixing the polymers in a porous inorganic matrix is widely considered a reliable solid composite electrolyte used in lithium batteries [33].

The limitations of the applications of current solid-state batteries mainly focus on addressing the interface between electrodes and solid electrolytes, as well as the low ionic conductivity of the electrolytes. At present, there are many types of electrolytes used in solid-state batteries, among which, solid polymer electrolytes include polythylene oxide (PEO), polyacrylonitrile (PAN) [34], and polyvinylidene fluoride hexafluoropropylene (PVDF-HFP) [35]. Polymer electrolytes have the advantages of low interfacial impedance, low cost, and easy processing, and they have enormous development potential and application prospects [36]. However, polymers are prone to crystallization at room temperature [37], leading to difficulties in ion transport and low ion conductivity, which cannot meet the requirements of practical applications. The composite electrolyte prepared by introducing inorganic fillers into the polymer shows several advantages such as good electrochemical stability and high ion conductivity [20]. Reported result have shown that the ionic conductivity of the composite electrolyte was 1.00 × 10^−5^ S·cm^−1^ by using Al_2_O_3_ as the filler and PEO as the matrix, while the ionic conductivity of the composite electrolyte material was 4.52 × 10^−4^ S·cm^−1^ when the nano-CeO_2_ filler was synthesized by electrospinning. The ionic conductivity of the composite electrolyte obtained by the combination of a SiO_2_ inorganic filler and PVDF-HFP is 2.71 × 10^−5^ S·cm^−1^, while the ionic conductivity of the composite solid electrolyte obtained by the combination of LLZO and PVDF-HFP is 1.76 × 10^−4^ S·cm^−1^. The addition of inorganic fillers is beneficial for the formation of electrolyte membranes, which can maintain integrity even at high temperatures, greatly improving the mechanical properties of polymer electrolytes [38]. Moreover, the inorganic fillers can not only reduce the glass transition temperature of a polymer electrolyte, but also interact with electrolyte salts and polymers to form an interfacial layer, greatly improving the migration rate of lithium ions.

In this study, Ta/Mo co-doped LLZO was synthesized by the solid-phase sintering method. With Li_6.4_La_3_Zr_1.45_Ta_0.5_Mo_0.05_O_12_ (LLZTMO) garnet powder as the filler [39] and low-cost PEO as the polymer matrix [40], the composite electrolyte battery was assembled and its electrochemical performance was analyzed. The prepared composite solid electrolytes have high ionic conductivity at 35 °C [41], a wide electrochemical window, and good interface stability with lithium anodes [42]. The full cell assembled with LiFePO4 as the positive electrode shows excellent rate performance and good cycle stability [43].

## 2. Experimental Methodology

### 2.1. Synthesis of LLZTMO–PEO

LiOH∙H_2_O (98%), La_2_O_3_ (99.99%), ZrO_2_ (99%), Ta_2_O_3_ (98%), MoO_3_ (98%), PEO, anhydrous acetonitrile, and LiTFSI were purchased from Shanghai Aladdin Biochemical Technology Co., Ltd. Preparation of LLZTMO was carried out according to the literature [44]. LiOH∙H_2_O, La_2_O_3_, ZrO_2_, Ta_2_O_3_, and MgO were weighed according to the stoichiometric ratio, then mixed well and dried in the vacuum oven overnight. After sintering at 900 °C for 12 h, and then ball milling for 12 h, the obtained LLZTMO powders were screened with a 400-mesh screen.

**Figure 1:**
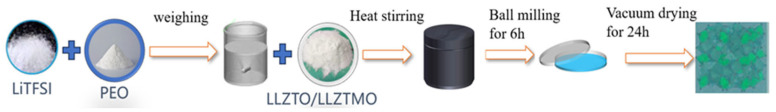
Schematic diagram of the composite electrolyte preparation process.

According to the molar ratio of Li^+^/EO (ethylene oxide) = 1/20, a proper amount of lithium salt (LiTFSI) was added into the PEO-containing beaker, followed by anhydrous acetonitrile, and stirring took place at 60 °C for 8 h. Then, LLZTMO was added into the beaker in a weight ratio of LLZTMO/PEO = 1/1, and milling continued for 6 h. After that, the resulting slurry was poured into a glass petri-dish mold, and after drying at 60 °C for 24 h, a uniformly dispersed and flat LLZTMO–PEO composite electrolyte was therefore gained. The preparation process is indicated in Figure 1. The preparation of the LLZTO–PEO composite electrolyte completely refers to the LLZTMO–PEO electrolyte but without adding a MoO_3_ precursor.

In order to prepare the cathode electrode, LiFePO_4_, Super P, and polyvinylidene fluoride (PVDF) were mixed with a weight ratio of 8:1:1, and then added into the 1-methyl-2-pyrrolidone (NMP) solvent. After stirring for 3 h, the slurry was coated on the aluminum foil homogenously and flatly, which was consequently dried in a vacuum oven at 120 °C for 12 h. Finally, the aluminum foil was cut into small circular pieces with a diameter of 13 mm, with a mass load of the cathode active materials of approximately 3 mg cm^−2^. All the CR2016 cells were assembled in an argon-filled glove box.

### 2.2. Characterizations and Measurements

Scanning electron microscopy (SEM, LEO1530 VP, Freising, Germany) was used to detect the surface morphology and microstructures of the samples. The crystal structure was investigated by a power X-ray diffractometer (XRD, Rigaku Ultima IV, Tokyo, Japan) using Cu Kα radiation (λ = 1.54056 Å) with a scanning rate of 10°/min. Thermogravimetric analysis (TGA) was performed on TG/TGA 6300 in air with a temperature range from 30 °C to approximately 800 °C and an increasing rate of 10 °C/min.

Cyclic voltammetry (CV) measurements were performed using an electrochemical workstation (CH Instruments, Bee Cave, TX, USA: CHI 660E) at a scan rate of 0.1 mV s^−1^ under a potential range from 4.2 to 2.4 V. The electrochemical impedance spectroscopy (EIS) was measured using DH7000C (DongHua Analytical, Jingjiang, China) with a frequency range from 0.01 Hz to 100 KHz and AC amplitude of 10 mV. The ionic conductivity (*σ*) of the solid electrolyte was determined by impedance spectroscopy based on the following equation:

*σ = L/R_b_S* (1)

where *R_b_* is the resistance of the bulk electrolyte, *L* is the thickness of the electrolyte film, and *S* is the surface area of the electrolyte.

The electrochemical stability of the solid electrolyte was detected using linear sweep voltammetry (LSV) with stainless steel as the working electrode and Li metal as the counting and reference electrodes. The voltage sweep window is between 2.0 and 6.0 V (vs. Li) at a scan rate of 1.0 mV s^−1^. Charge/discharge tests of symmetric Li/LLZTMO–PEO/Li and Li/LLZTO–PEO/Li batteries were conducted at room temperature and different current densities. The electrochemical performance test of LiFePO_4_/LLZTMO–PEO/Li and LiFePO_4_/LLZTO–PEO/Li full cells was carried out at a voltage range of 4.2 to 2.5 V at room temperature with a charge/discharge rate of 0.2C (1C = 170 mAh/g).

## 3. Results and Discussion

### 3.1. SEM

**Figure 2:**
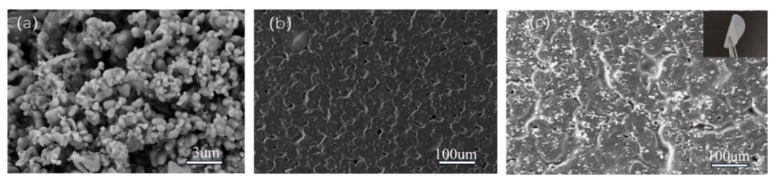
The SEM images of the LLZTMO powders (**a**), PEO electrolyte (**b**), and the LLZTMO–PEO composite solid electrolyte (**c**) with 50%wt LLZTMO; inset in (**c**) is the photo of the composite electrolyte film (LLZTO and LLZTO–PEO show almost the same SEM images as LLZTMO and LLZTMO–PEO).

Figure 2 shows the SEM images of the as-prepared LLZTMO powders, PEO film, and the LLZTMO–PEO composite electrolyte. The size of the LLZTMO powders varies from nanometers to micrometers based on the solid-state reactions and ball milling. As can be seen in Figure 2a, LLZTMO contains uniform spherical particles, which can reduce the path of lithium-ion transport; in addition, the spherical particles can also make good contact with the PEO matrix, thereby reduce the impedance of the solid electrolyte. In Figure 2b, the PEO film is porous and wrinkled. As can be seen from Figure 2c, when LLZTMO was added into the PEO slurry, the LLZTMO powders are evenly distributed on/in the PEO film due to the ball milling treatment. The uniform distribution of inorganic fillers helps to provide a uniform lithium-ion transport channel and consequently improve the ion transport rate. The inset of Figure 2c is the optical photo of the prepared LLZTMO–PEO electrolyte film, which is white, flexible, and translucent. The LLZTO powders and the LLZTO–PEO composite electrolyte show almost same SEM images as LLZTMO powders and LLZTO–PEO.

### 3.2. XRD

**Figure 3:**
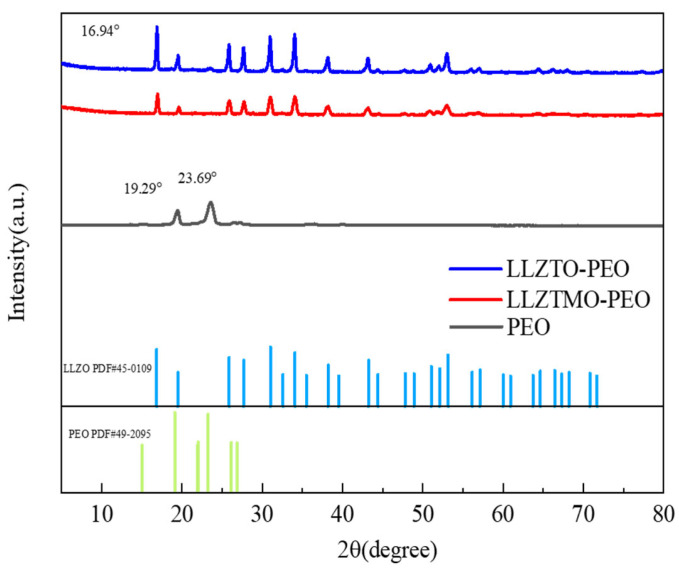
The XRD patterns of PEO, and LLZTO–PEO and LLZTMO–PEO composites.

The XRD pattern of the composite solid electrolyte and PEO is shown in Figure 3. PEO shows the characteristic peaks appearing at 19.29° and 23.69°, which are very consistent with the previous reports [45] and the stand card PDF#49-2095. It can be seen that the positions of the diffraction peaks and the relative intensity of each peak of LLZTO and LLZTMO are highly accordant with that of the garnet-type Li_5_La_3_Nb_2_O_12_ (standard card PDF#45-0109). There are no other miscellaneous phases found, indicating that the synthesized LLZTO/LLZTMO is a cubic-phase garnet-type structure. The positions of the diffraction peaks at 16.84°, 25.84°, 27.66°, 31.00°, 34.05°, 38.21°, 43.23°, and 53.13° correspond to (211), (101), (400), (420), (422), (501), (611), and (642) crystal faces [46]. The composite electrolytes show full XRD peaks of both garnet and PEO components, which means that the presence of LLZTO/LLZTMO does not affect the crystallization of PEO. But, if carefully comparing, one would find that the XRD line of PEO at 23.69° shifts to a higher degree, which may be due to the presence of LLZTO/LLZTMO increasing the stress of PEO or reducing the layer spacing of PEO.

### 3.3. TGA

**Figure 4:**
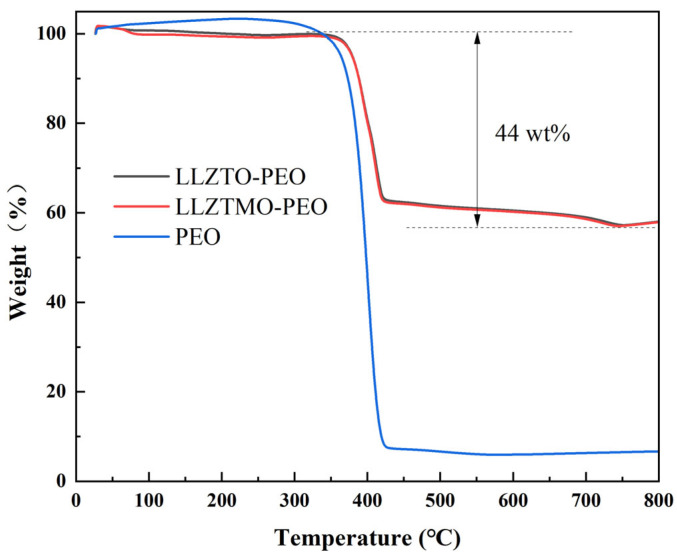
Thermogravimetric analysis of the PEO LLZTO–PEO and LLZTMO–PEO samples.

In order to obtain the information on the composition, component content, and thermal stability of LLZTO–PEO and LLZTMO–PEO electrolytes, a TGA test was conducted on the prepared composite electrolyte (repeat three times), as shown in Figure 4. The thermogravimetric curves of LLZTO–PEO and LLZTMO–PEO showed almost similar trends, and the weight loss of both electrolytes was also same. When the temperature is below 100 °C, the evaporation of water accounts for about 3% of the total mass. In the temperature range of 150 °C to 400 °C, the weight of the two electrolytes remained stable, indicating that there was no significant mass loss at this stage. However, when the temperature rose to 410 °C and above, the quality of both electrolyte films showed a sharp decline, which is because PEO and the lithium salt LiTFSI decompose under high temperatures. In particular, the curves show that the decomposition rate of the electrolyte reaches its highest at 413 °C. When the temperature is close to 453 °C, only a small amount of the main decomposition products of PEO remains, after which the thermogravimetric curve gradually flattens out until the temperature rises to 740 °C; the remaining mass of LLZTO–PEO and LLZTMO–PEO composite solid electrolytes is about 56% of the initial mass. It can be seen from the results of thermogravimetric analysis that the garnet-type structure fillers such as LLZTO and LLZTMO show excellent thermal stability at high temperatures, as they can absorb the heat at high temperatures and thus strengthen the PEO matrix, thereby improving the safety performance of the battery. The final residual mass of these two composite electrolytes is mainly composed of LLZTO or LLZTMO ceramic particles, accounting for about 56% of the total residual mass fraction. Moreover, the thermal decomposition curve of PEO shows that the decomposition begins below 400 °C, while the thermal decomposition of LLZTO–PEO and LLZTMO–PEO composite solid electrolyte films mainly occurs after 413 °C, and the decomposition slope decreases to a certain extent, indicating that the addition of LLZTO/LLZTMO also increases the decomposition temperature of PEO.

### 3.4. Ionic Conductivity and Activation Energy

**Figure 5:**
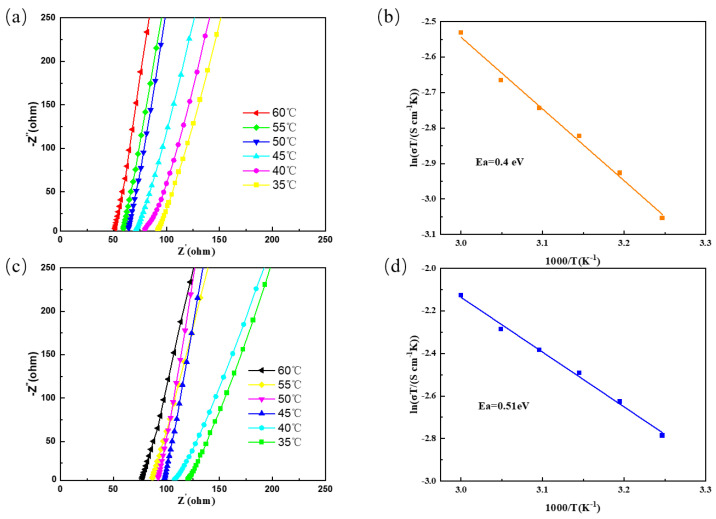
(**a**) LLZTMO–PEO variable temperature impedance; (**b**) LLZTMO–PEO corresponds to the activation energy; (**c**) LLZTO–PEO variable temperature impedance; (**d**) LLZTO–PEO corresponds to the activation energy.

**Figure 6:**
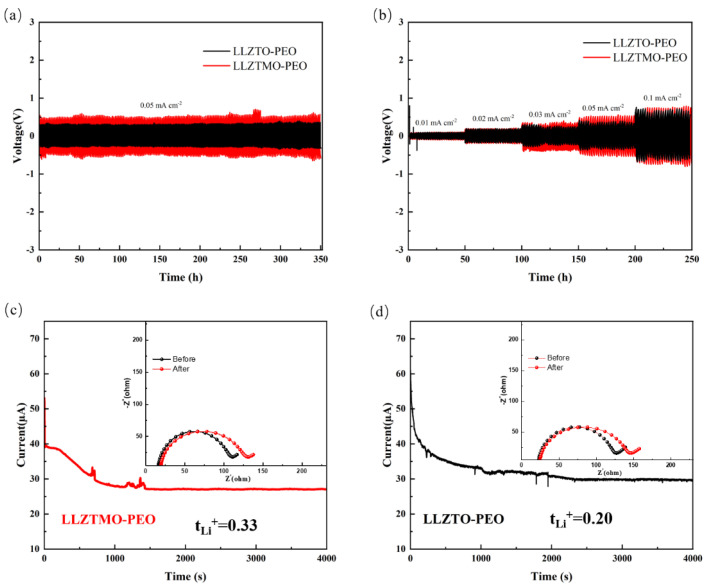
(**a**) Symmetrical battery constant current charge and discharge cycle; (**b**) Multiplier test curve for symmetrical batteries line; (**c**) i-t polarization curve of LLZTMO–PEO electrolyte and the symmetric battery impedance before and after polarization; (**d**) i-t polarization curve of LLZTO–PEO electrolyte and the symmetric battery impedance before and after polarization.

The ionic conductivity of the garnet composite solid electrolytes synthesized with LLZTMO/LLZTO as a filler was studied by testing the impedance change in the temperature range of 35~60 °C, and the trend of change with temperature and corresponding EIS diagram are shown in Figure 5a,c. In the entire temperature range, the EIS diagrams of LLZT–PEO and LLZTMO–PEO have only one straight line, indicating that the synthesized composite solid electrolyte is similar to the gel electrolyte, which is conducive to lithium ions. As is shown in Figure 5a, during the temperature change of 35 °C~60 °C, the impedance of LLZTMO–PEO decreases from 91.71 Ω at 35 °C to 51.27 Ω at 60 °C, and the ionic conductivity at 35 °C is 2.00 × 10^−4^ S·cm^−1^ and at 60 °C is 3.58 × 10^−4^ S·cm^−1^. As is shown in Figure 6c, during the temperature change of 35 °C~60 °C, the impedance of LLZTO–PEO decreases from 119.96 Ω at 35 °C to 76.9 Ω at 60 °C, and the ionic conductivity at 35 °C is 1.53 × 10^−4^ S·cm^−1^ and at 60 °C is 2.39 × 10^−4^ S·cm^−1^. LLZTMO can further optimize the transmission path of lithium ions. In addition, the introduction of LLZTO/LLZTMO fillers brings high ionic conductivity, as the ionic conductivity of PEO is in the range of 10^−7^~10^−6^ S·cm^−1^. Consequently, the fillers can effectively cross-link the polymerization in the PEO matrix, avoiding the aggregation problem that may be caused by inorganic fillers. The high specific surface area and rich pore structure of the nanoparticles also facilitate the dissolution of lithium salts, and thus help to prevent the crystallization of PEO. Therefore, an efficient lithium-ion transport channel is formed in the entire system, which significantly improves the conduction efficiency.

According to the Nernst–Einstein formula, the ion migration apparent activation energy of the two flexible composite electrolyte membranes was calculated, and the results are shown in Figure 5b,d. The apparent activation energy of LLZTMO–PEO is only 0.40 eV, which is lower than that of LLZTO–PEO (0.51 eV), indicating that the energy barrier of lithium-ion migration in LLZTMO–PEO is lower. This is consistent with results demonstrating that LLZTMO–PEO has higher ionic conductivity than LLZTO–PEO.

### 3.5. Lithium Compatibility Analysis

In the constant current cycling experiment, a symmetric Li|LLZTMO/LLZTO–PEO|Li cell was prepared to evaluate the interface stability between LLZTMO–PEO and Li metal electrodes, as well as to study the mechanical stability for lithium ions dendritic growth. Periodic charging and discharging were carried out at a constant current density to simulate lithium stripping and electroplating processes at room temperature. The voltage curve over time during the cyclic test process is shown in Figure 6a. Stable voltage can be obtained over 350 h, indicating that the composite solid electrolyte has good compatibility with metallic lithium and forms a stable electrolyte/Li interphase. The rate performance of the Li|LLZTMO/LLZTO–PEO|Li symmetric cell under different current densities was investigated, as shown in Figure 6b. The results indicate that when the current density increases to 0.1 mA cm^−2^, the cell continued to run steadily for 250 h. Both of them exhibit a very stable polarization voltage. The results show that reversible lithium plating and stripping of lithium metal can be achieved.

Migration number is an index to measure the transfer efficiency of lithium ions in an electrolyte, and it is also a key parameter to evaluate the performance of an electrolyte. The lithium-ion migration number of LLZTO–PEO composites is 0.20 by analyzing the i-t polarization curve. However, for LLZTMO, the value increases to 0.33. This suggests that a larger proportion of freely moving lithium ions are present in LLZTMO–PEO. This effect promotes the dissociation of lithium salts, increases the concentration of free lithium ions, and ultimately leads to an increase in the migration number of lithium ions in the composite electrolyte. The increase in the number of lithium-ion migrations further optimizes the contact interface stability between the electrolyte and the lithium metal negative electrode.

### 3.6. CV, LSV, and EIS

**Figure 7:**
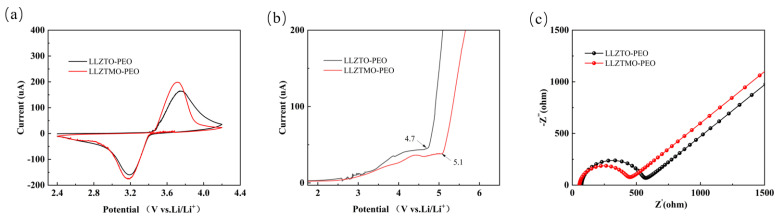
(**a**) Li|LLZTMO/LLZTO–PEO|LiFePO_4_ CV curve (0.1 mV s^−1^); (**b**) Li|LLZTMO/LLZTO–PEO|SS LSV curve (1.0 mV s^−1^); (**c**) Li|LLZTMO/LLZTO–PEO|LFP impedance diagram.

Figure 7a shows the CV curves of LLZTO/LLZTMO–PEO-based batteries in the voltage range from 2.4 V to 4.2 V, with a scanning rate of 0.1 mV s^−1^. Both cells show significant reduction peaks at ~3.2 V and strong oxidation peaks at ~3.8 V, corresponding to the lithium plating and stripping processes of lithium ions. With careful observation, one may find that the CV curve of LLZTMO–PEO has large REDOX peak areas, indicating that it has a large specific capacity. According to Figure 7b, we can observe that under the test environment of 60 °C, the SS/LLZTO–PEO/Li cell begins to show a significant increase in current at 4.7 V, which marks the beginning of electrolyte decomposition. As the voltage continues to rise during the test, the current released by the decomposition reaction continues to increase. For the LLZTMO–PEO composite electrolyte, the initial voltage of oxidation decomposition is increased to 5.1 V. This change may be due to the interaction between LLZTMO and the polymer matrix interface, resulting in a change in the electronic energy level of PEO, thereby improving the antioxidant stability of PEO. To explore the charge transfer impedance of the battery, EIS measurements were performed on the half battery, as shown in Figure 7c. Each EIS curve consists of a semicircle caused by charge transfer resistance (Rct) [47] and a diagonal line corresponding to lithium-ion diffusion. It can be seen that the charge transfer impedance of LLZTO–PEO is 505.09 Ω, and the interface charge transfer impedance of LLZTMO–PEO is 397.13 Ω [48].

### 3.7. Electrochemical Performance of Li|LLZTMO/LLZTO–PEO|LiFePO_4_

**Figure 8:**
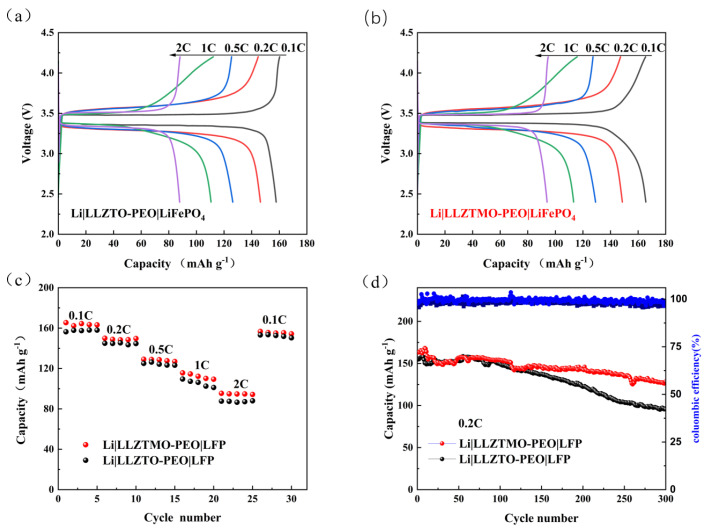
(**a**) Li|LLZTO–PEO|LiFePO_4_ and (**b**) Li|LLZTMO–PEO|LiFePO_4_ full cell polarization trends; (**c**) rate performance test; and (**d**) cycling properties at 0.2C of the Li|LLZTO/LLZTMO–PEO|LiFePO_4_ full cells.

In order to better evaluate the electrochemical performance of the solid-state full cells, the polarization trend, rate performance, and cycle stability have been investigated, as shown in Figure 8. As is shown in Figure 8a,b, comparing the constant current charge and discharge curves of Li|LLZTO–PEO|LFP and Li|LLZTMO–PEO|LFP batteries, it can be seen that the polarization overpotential of the battery is small, the battery charge and discharge are stable, and the polarization increases slowly with current density, indicating that there are basically no other side reactions during the process (Table 1). The polarization potential of Li|LLZTMO–PEO|LFP cells is lower than that of Li|LLZTO–PEO|LFP cells, which corresponds to a lower lithium-ion migration barrier, and is consistent with the results of the whole-battery EIS test.

The rate performance of the full cell at different current densities from 0.1 to 2C at room temperature 25 °C is shown in Figure 8c. At a 0.1C current density, the specific discharge capacities of the assembled batteries are 156.4 mAh g^−1^ and 165.4 mAh g^−1^ for LLZTO and LLZTMO electrolytes, respectively. After cycling 0.2, 0.5, 1.0, and 2C cycles and recovery to 0.1C, the specific discharge capacity of the battery recovered to 150.4 mAh g^−1^ and 156.7 mAh g^−1^, respectively, indicating that the battery assembled by the composite solid electrolyte has good reversible performance. The cycling stability test is shown in Figure 8d at 0.2C. It can be seen that the initial specific discharge capacities of LLZTO–PEO and LLZTMO–PEO are 145.6 and 157.5 mAh g^−1^, respectively. After 300 cycles, the specific discharge capacities are 102.6 and 127.0 mAh g^−1^, and the capacity retention rates are 70.5 and 80.6%, respectively. The results show that LLZTMO–PEO cells have a higher specific discharge capacity, which is due to the fact that Mo ion doping accelerates the relative motion of lithium ions and promotes the migration of lithium ions. In conclusion, the electrochemical test results indicated that the cell with LLZTMO–PEO composite solid-state electrolyte exhibited better rate performance, high reversible capacity, and a good cycle life, compared with the LLZTO–PEO electrolyte.

## 4. Conclusions

Solid composite electrolytes with PEO as the matrix and LLZTMO/LLZTO as the fillers have been synthesized. The experimental results show that the presence of Mo in the garnet-type oxide can further increase its ionic conductivity and reduce the interface resistance between the electrolyte and electrode, thus improving the electrochemical performance of symmetric or full batteries. The LLZTMO–PEO composite solid electrolyte has a high ionic conductivity of 2.00 × 10^−4^ S·cm^−1^ at 35 °C, a wide electrochemical stability window of 5.1 V, and a lithium-ion migration number of 0.33. The initial specific discharge capacity of the Li|LLZTMO–PEO|LiFePO_4_ battery could reach 157.5 mAh g^−1^, and the specific discharge capacity is still 127.0 mAh g^−1^ after 300 cycles with the capacity retention rate of 80.6%. The results showed that the co-doped LLZTMO solid electrolyte has a potential application prospect in solid-state lithium-ion batteries, whose electrochemical properties are comparable to the commercial liquid-electrolyte lithium-ion batteries.

## Figures and Tables

**Table 1:** Power density and energy density comparison.

Rate C	Li|LLZTMO–PEO|LiFePO_4_ (mAh g^−1^)	Li|LLZTO–PEO|LiFePO_4_ (mAh g^−1^)
0.1C	165.4	156.4
0.2C	149.9	144.9
0.5C	129.1	125.0
1C	115.6	109.5
2C	94.3	87.6
0.1C	156.7	150.4

## Data Availability

Data are contained within the article.
